# The implementation of artificial intelligence in upper extremity surgery: a systematic review

**DOI:** 10.3389/frai.2025.1621757

**Published:** 2025-09-29

**Authors:** Dylan Parry, Brennon Henderson, Paul Gaschen, Diane Ghanem, Evan Hernandez, Anceslo Idicula, Tammam Hanna, Brendan MacKay

**Affiliations:** ^1^School of Medicine, Texas Tech University Health Sciences Center, Lubbock, TX, United States; ^2^Department of Orthopaedic Surgery, Texas Tech University Health Sciences Center, Lubbock, TX, United States; ^3^Department of Orthopaedic Surgery, The Johns Hopkins Hospital, Baltimore, MD, United States; ^4^Department of Health Sciences, College of Health Sciences, Rush University, Chicago, IL, United States; ^5^Department of Hand and Microvascular Surgery, University Medical Center, Lubbock, TX, United States

**Keywords:** artificial intelligence, machine learning, orthopedics, surgery, upper extremity

## Abstract

**Introduction:**

The rapid expansion of artificial intelligence (AI) in medicine has led to its increasing integration into upper extremity (UE) orthopedics. The purpose of this systematic review is to investigate the current landscape and impact of AI in the field of UE surgery.

**Methods:**

Following PRISMA (Preferred Reporting Items for Systematic Reviews and Meta-Analyses) guidelines, a systematic search of PubMed was conducted to identify studies incorporating AI in UE surgery. Review articles, letters to the editor, and studies unrelated to AI applications in UE surgery were excluded.

**Results:**

After applying inclusion/exclusion criteria, 118 articles were included. The publication years ranged from 2009 to 2024, with a median and mode of 2022 and 2023, respectively. The studies were categorized into six main applications: automated image analysis (36%), surgical outcome prediction (20%), measurement tools (14%), prosthetic limb applications (14%), intraoperative aid (10%), and clinical decision support tools (6%).

**Discussion:**

AI is predominantly utilized in image analysis, including radiograph and MRI interpretation, often matching or surpassing clinician accuracy and efficiency. Additionally, AI-powered tools enhance the measurement of range of motion, critical shoulder angles, grip strength, and hand posture, aiding in patient assessment and treatment planning. Surgeons are increasingly leveraging AI for predictive analytics to estimate surgical outcomes, such as infection risk, postoperative function, and procedural costs. As AI continues to evolve, its role in UE surgery is expected to expand, improving decision-making, precision, and patient care.

## Introduction

Artificial Intelligence (AI) refers to computational algorithms that model human intelligence in learning, decision-making, and problem-solving. In recent years, the application of AI in healthcare has exponentially increased, driven by advancements in machine learning models, increased computing power, and improved data availability. The development of sophisticated AI systems, such as ChatGPT and deep learning algorithms, has enhanced accessibility for healthcare professionals, patients, and researchers. Prior studies have shown the diverse applications of AI in medicine, including image recognition for fracture detection and classification, preoperative risk assessment, clinical decision support, and predictive modeling of treatment outcomes (Myers et al., 2020; Langerhuizen et al., 2019).

Due to the rapid expansion of AI implementation in medicine in recent years, AI is being used in more areas and more accurately than ever before, including in upper extremity (UE) orthopedics. A 2019 systematic review of 12 studies on AI-driven fracture detection in general orthopedics highlighted a promising performance with near-perfect prediction in five articles (AUC 0.95–1.0) (Langerhuizen et al., 2019). This near-perfect accuracy provided some insight into the capabilities of AI in advancing modern medicine and aiding clinicians in their work, especially as updated AI models continue to rise.

A scoping review by Keller et al. (2023) examined AI applications in hand surgery before April 2021, revealing limited utilization compared to other medical specialties). Given the rapid advancements since then, this systematic review aims to comprehensively assess the current landscape of AI in UE surgery. By analyzing the existing body of evidence, we seek to elucidate the potential clinical impacts of AI technologies and identify key areas for future research and development within this important field of UE orthopedics.

## Materials and methods

### Study search strategy

This systematic review was conducted in accordance with the Preferred Reporting Items for Systematic and Meta-Analysis (PRISMA) (Tricco et al., 2018) guidelines, ensuring methodological transparency and accuracy. A comprehensive literature search was performed using the MEDLINE/PubMed database. The search focused on identifying relevant literature pertaining to the use of AI in UE surgery. The search strategy was designed to capture all relevant studies published between November 2009 and April 2024. The electronic search strategy used was: *(Artificial Intelligence OR Machine Learning OR Deep Learning) AND (Diagnosis OR Detection) AND (Hand Surgery OR Arm Surgery OR Elbow Surgery OR Shoulder Surgery).*

### Inclusion and exclusion criteria

Studies were included if they evaluated AI applications in UE surgery and were original research articles. Excluded studies included those unrelated to AI in UE surgery, review articles, letters to the editor, conference abstracts, and articles not published in English.

### Selection process

All database search results were imported into Rayyan, a systematic review management tool, where duplicates were automatically removed using a trained AI system, as described by Adu et al. (2024). Two independent reviewers then performed an initial screening of titles and abstracts to exclude studies that did not meet the eligibility criteria. Subsequently, full-text articles of potentially relevant studies were then reviewed independently by both reviewers. At any point, any disagreements regarding study inclusion were resolved through discussion, with the corresponding author serving as the final adjudicator in cases of unresolved discrepancies. Included studies were then sorted into categories based on the perceived primary focus of the paper. When study overlap between two categories occurred, discussion took place, and the studies were placed into their perceived primary category.

## Results

The initial literature search generated 1,097 unique articles, of which 118 met the inclusion criteria after abstract review and application of the exclusion criteria. No sources were included from grey literature or non-PubMed sources.

**Figure 1:**
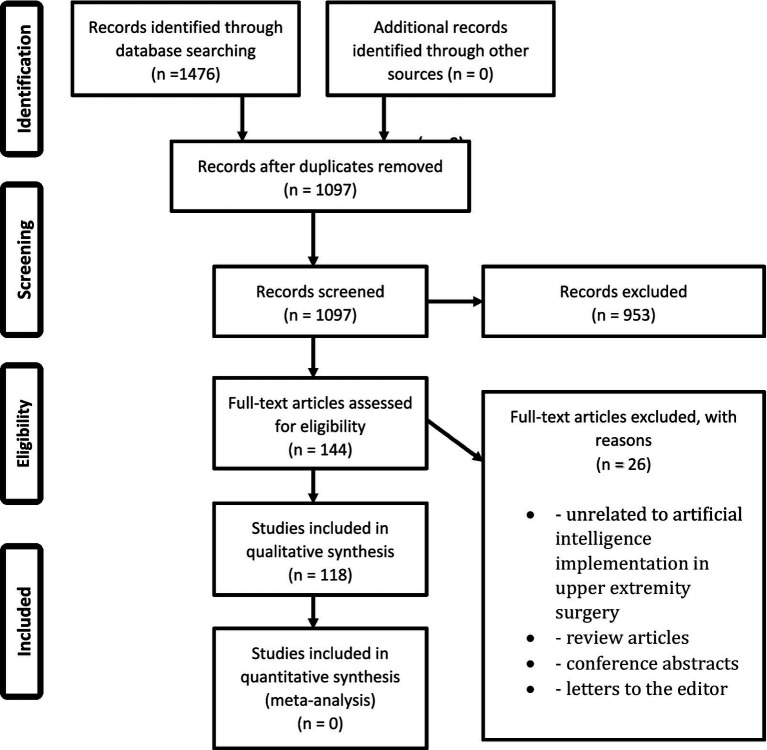
PRISMA flowchart. Represents the preferred reporting items for systematic and meta-analysis (PRISMA) flowchart for identification, screening, and eventual inclusion of articles in this study.

**Figure 2:**
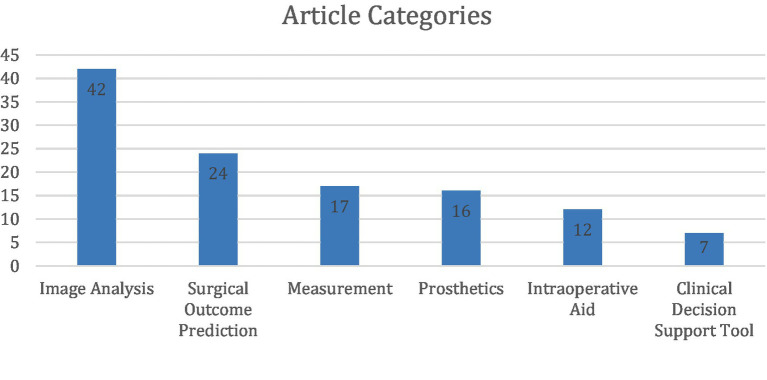
Distribution of 118 studies across 6 categories, with counts derived from non-overlapping classifications after consensus.

These studies were categorized into six primary areas of AI implementation in upper extremity (UE) surgery: automated image analysis (36%), surgical outcome prediction (20%), measurement tools (14%), prosthetic limb applications (14%), intraoperative assistance (10%), and clinical decision support tools (6%) (Figures 1, 2).

### Study overlap

**Figure 3:**
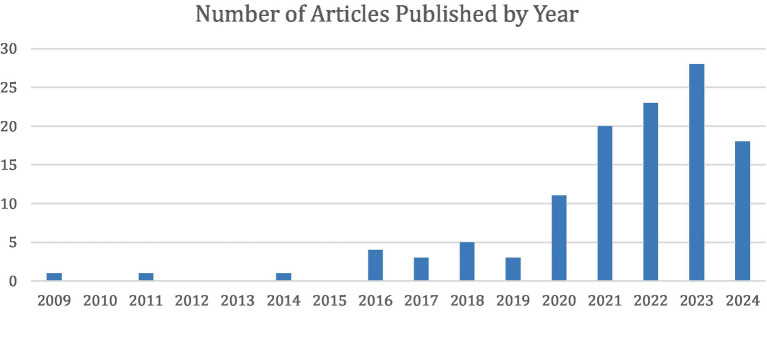
Distribution of 118 studies stratified by year of publication.

**Figure 4:**
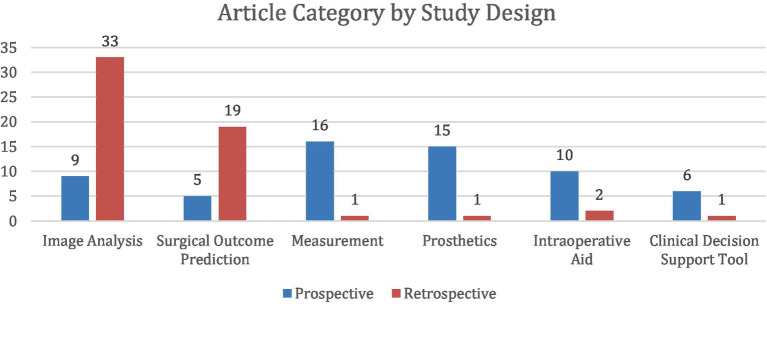
Distribution of 118 studies stratified into 6 categories and further divided by study design. Prospective studies were classified as those in which participants were followed forward in time from the point of the study’s initiation. Retrospective studies were classified as those in which researchers examined existing records of past events to find associations between exposures and outcomes. Any disagreements on study design were resolved through discussion, with the corresponding author serving as the final adjudicator in cases of unresolved discrepancies.

Following categorization, 11 of the 118 studies had overlap between two categories. Seven (Minelli et al., 2022; Ro et al., 2021; Alike et al., 2023; Lee et al., 2024; Gu et al., 2022; Kim et al., 2021; Ramkumar et al., 2018) of the studies overlapped between the Image Analysis and Measurement categories. Two (Kluck et al., 2023; Lu et al., 2021) of the studies overlapped between Image Analysis and Surgical Outcome Prediction. One study (Lee et al., 2018) overlapped between Image Analysis and Intraoperative Aid. One study (Cheng et al., 2023) overlapped between Intraoperative Aid and Clinical Decision Support Tool (Figures 3, 4).

### Automated image analysis

Similar to a prior review on hand surgery, the most common application of AI in UE surgery was automated image analysis (Keller et al., 2023), accounting for 42 articles (Anttila et al., 2023; Chung et al., 2018; Dipnall et al., 2022; Droppelmann et al., 2022; Guermazi et al., 2022; Guo et al., 2023; Hahn et al., 2022; Minelli et al., 2022; Ro et al., 2021; Yi et al., 2020; Anttila et al., 2022; Feuerriegel et al., 2023; Feuerriegel et al., 2024; Grauhan et al., 2022; Kang et al., 2021; Kim et al., 2022; Shinohara et al., 2023; Wei et al., 2022; Yang et al., 2024; Yoon et al., 2023; Zech et al., 2024; Zech et al., 2023; Alike et al., 2023; Alike et al., 2023; Benhenneda et al., 2023; Jopling et al., 2021; Keller et al., 2023; Kuok et al., 2020; Lee et al., 2024; Lee et al., 2023; Mert et al., 2024; Ni et al., 2024; Oeding et al., 2024; Shinohara et al., 2022; Suzuki et al., 2022; Anderson et al., 2023; Cirillo et al., 2019; Georgeanu et al., 2022; Jeon et al., 2023; Li and Ji, 2021; Cirillo et al., 2021; Yoon and Chung, 2021). These studies focused on AI-driven interpretation of radiographs, magnetic resonance imaging (MRI), ultrasound, and arthroscopic images, with radiographs being the most frequently analyzed modality.

Assessing the implementation of AI in examining radiographs accounted for 24 articles (Anttila et al., 2023; Chung et al., 2018; Dipnall et al., 2022; Guermazi et al., 2022; Minelli et al., 2022; Yi et al., 2020; Anttila et al., 2022; Grauhan et al., 2022; Kang et al., 2021; Wei et al., 2022; Yang et al., 2024; Yoon et al., 2023; Zech et al., 2024; Zech et al., 2023; Alike et al., 2023; Alike et al., 2023; Jopling et al., 2021; Keller et al., 2023; Lee et al., 2024; Mert et al., 2024; Suzuki et al., 2022; Anderson et al., 2023; Jeon et al., 2023; Yoon and Chung, 2021). AI models show promising capability by quickly and accurately detecting fractures (clavicle, arm, elbow, wrist, hand), measuring critical shoulder angle, identifying shoulder arthroplasty models, and detecting conditions such as enchondromas, joint dislocations, rotator cuff tendon tears, and scapholunate ligament ruptures.

Six studies (Chung et al., 2018; Guo et al., 2023; Zech et al., 2023; Mert et al., 2024; Ni et al., 2024; Suzuki et al., 2022) directly compared AI performance to human clinicians in image analysis, showing that AI matched or outperformed human readers in diagnostic accuracy and speed. One study demonstrated that an AI model achieved an accuracy of 99.3%, a sensitivity of 98.7%, and a specificity of 100% in detecting distal radius fractures, surpassing the performance of three hand orthopedic surgeons (Suzuki et al., 2022). In detecting proximal humerus fractures, AI also outperformed general physicians and non-specialized orthopedists, particularly in complex 3- and 4-part fractures (Chung et al., 2018). AI models integrating deep visual features with clinical data improved diagnostic accuracy for supraspinatus/infraspinatus tendon complex (SITC) injuries, significantly benefiting junior physicians with limited experience (Alike et al., 2023).

A separate study showed that the diagnostic accuracy of an AI algorithm on dorsopalmar radiography regarding scapholunate ligament integrity was close to that of the experienced human reader (e.g., differentiation of Geissler’s stages ≤ 2 versus > 2 with a sensitivity of 74% and a specificity of 78% compared to 77 and 80%) with a correlation coefficient of 0.81 (*p* < 0.01) (Keller et al., 2023). When AI and humans’ ability to analyze radiographs were directly compared to each other in terms of accuracy or speed, we did not identify any articles that showed humans significantly outperforming AI. Table 1 shows the results of each study that directly compared the image analysis performance between AI models and human readers.

**Table 1:** The results of image analysis when various AI models were directly compared to human readers.

Study*	Task	Dataset	AUC^†^	Accuracy	Sensitivity	Specificity
Guo et al. (2023)	Detect supraspinatus tears (MRI)	770 MRIs	- - - - -	0.870 (AI) 0.891 (senior surgeon) 0.761 (junior surgeon) 0.862 (senior radiologist) 0.775 (junior radiologist)	0.913 (AI) 0.935 (senior surgeon) 0.913 (junior surgeon) 0.935 (senior radiologist) 0.891 (junior radiologist)	0.848 (AI) 0.870 (senior surgeon) 0.685 (junior surgeon) 0.826 (senior radiologist) 0.717 (junior radiologist)
Chung et al. (2018)	Detect and classify proximal humerus fractures (X-ray)	1,891 images (1 per person) of normal shoulders (n = 515) and 4 proximal humerus fracture types (greater tuberosity, 346; surgical neck, 514; 3-part, 269; 4-part, 247) classified by 3 specialists were evaluated	- - - -	0.96 (AI) 0.85 (general physician) 0.93 (general orthopedist) 0.93 (shoulder orthopedist)	0.99 (AI) 0.82 (general physician) 0.93 (general orthopedist) 0.96 (shoulder orthopedist)	0.97 (AI) 0.94 (general physician) 0.97 (general orthopedist) 0.98 (shoulder orthopedist)
Zech et al. (2023)	Detect a range of pediatric UE fractures (X-ray)	58,846 UE X-rays (finger/hand, wrist/forearm, elbow, humerus, shoulder/clavicle) from 14,873 pediatric and young adult patients	- -	0.897 (AI) 0.851 (residents)	0.908 (AI) -	0.887 (AI) -
Mert et al. (2024)	Detect distal radius fractures (X-ray)	100 wrist X-rays with and 50 without distal radius fractures of patients who had received X-rays due to suspected fracture	0.93 (ChatGPT) 0.985 (hand surgery resident) 0.85 (medical student) 0.99 (gleamer bone view)	- - - -	0.88 (ChatGPT) 0.99 (resident) 0.98 (student) 1.00 (gleamer bone view)	0.98 (ChatGPT) 0.98 (resident) 0.72 (student) 0.98 (gleamer bone view)
Ni et al. (2024)	Detect SLAP lesions (MRI)	636 patients (SLAP lesions confirmed via shoulder arthroscopy)	0.98 (AI) - - -	0.96 (AI) 0.85 (radiologist 15) 0.83 (radiologist 10) 0.81 (radiologist 7)	0.94 (AI) 0.91 (radiologist 15) 0.81 (radiologist 10) 0.78 (radiologist 7)	1.00 (AI) 0.76 (radiologist 15) 0.85 (radiologist 10) 0.85 (radiologist 7)
Suzuki et al. (2022)	Detect distal radius fractures (X-ray)	961 (1971 total images)	- - - -	0.993 (AI) 0.973 (surgeon 1) 0.947 (surgeon 2) 0.967 (surgeon 3)	0.987 (AI) 0.960 (surgeon 1) 0.960 (surgeon 2) 0.960 (surgeon 3)	1.00 (AI) 0.987 (surgeon 1) 0.933 (surgeon 2) 0.973 (surgeon 3)

AUC, Area Under Curve; MRI, Magnetic Resonance Imaging; AI, Artificial Intelligence; SLAP, Superior labrum anterior–posterior; UE, Upper Extremity. ^†^A dash (−) indicates that these specific data were not available in the study. Studies are listed by the last name of the first author.

Additionally, several studies (Guermazi et al., 2022; Yoon et al., 2023; Zech et al., 2024; Alike et al., 2023; Anderson et al., 2023) evaluated AI-assisted human image analysis and found that AI augmentation improved clinician accuracy. In a retrospective study of fracture detection, AI-assisted readings increased sensitivity by 10.4% (75.2% vs. 64.8%), while maintaining specificity and reducing average reading time by 6.3 s per case (Guermazi et al., 2022). One study showed AI improves fracture detection among radiology and orthopedic residents in both pediatric and adult patients (Zech et al., 2024). Additionally, this study shows that AI enhances the specificity, sensitivity, and accuracy of physicians diagnosing supraspinatus/infraspinatus tendon complex injuries (Alike et al., 2023). Furthermore, AI assistance was shown to improve physician diagnostic sensitivity and specificity as well as interobserver agreement for the diagnosis of occult scaphoid fractures (Yoon et al., 2023). Similar findings were shown in several specialties, such as orthopedics, emergency medicine, radiology, and primary care, where the fracture miss rate was significantly reduced when aided by AI (Anderson et al., 2023).

### Surgical outcome prediction

A total of 24 articles (Allen et al., 2024; Biron et al., 2020; Digumarthi et al., 2024; Giladi et al., 2023; Gowd et al., 2019; Gowd et al., 2022; Hoogendam et al., 2022; Karnuta et al., 2020; Kausch et al., 2020; King et al., 2023; Kluck et al., 2023; Kumar et al., 2021; Kumar et al., 2020; Kumar et al., 2022; Li et al., 2023; Lu et al., 2022; Lu et al., 2021; Mclendon, 2021; Oeding et al., 2023; Polce et al., 2021; Rajabzadeh-Oghaz et al., 2024; Roche et al., 2021; Shinohara et al., 2024; Simmons et al., 2023; Vassalou et al., 2022) investigated AI’s ability to predict surgical outcomes in UE surgery. These studies focused on rotator cuff arthropathy, carpal tunnel syndrome, and calcific tendonitis, with total shoulder arthroplasty (TSA) being the most frequently analyzed procedure. Among these, 10 studies specifically assessed AI’s ability to predict patient outcomes following anatomic (ASA) or reverse (RSA) total shoulder arthroplasty. All articles except one were retrospective and tested a variety of language learning models (LLMs) with different input variables.

AI models demonstrated high predictive accuracy in estimating postoperative outcomes, such as improvements in shoulder function, patient satisfaction, and complication risk. The predictive variables analyzed included patient history/demographics, pain and functionality scores, physical exam findings, imaging data (X-ray, CT), and laboratory values. Multiple studies showed that machine learning models could achieve AUC values between 0.71 and 0.94, effectively predicting postoperative range of motion (ROM), risk of infection, and the likelihood of requiring revision surgery. One study demonstrated 92.9% accuracy (AUC 0.875) in predicting multiple clinical outcomes after TSA using a limited set of 19 preoperative variables, minimizing the need for extensive data input (Kumar et al., 2020).

For each of the 10 studies involving total shoulder arthroplasty patients, Table 2 details the input variables used, data set size, predictive task, and predictive ability.

**Table 2:** The predictive task, utilized input variables, dataset, and predictive ability of the 10 studies involving total shoulder arthroplasty of the 24 that discussed the ability of AI to predict surgical outcomes.

Study	Type	Predictive task	Input variables	Dataset*	Predictive ability^†^
McLendon (2021)	Retrospective	Improvement in ASES Score	History / Demographics Questionnaires Imaging Results	472 (431 ASA, 41 RSA)	Sensitivity - 0.94^‡^
Kumar et al. (2022)	Retrospective	Improvement in Internal Rotation	History / Demographics Questionnaires Physical Exam Findings Imaging Results	6,468 (2,270 ASA, 4,198 RSA)	AUC - 0.79^§^ Accuracy - 82%^§^
Kumar et al. (2021)	Retrospective	Improvement in Multiple Clinical Outcomes^¶^	History / Demographics Questionnaires Physical Exam Findings	5,774 (2,153 ASA, 3,621 RSA)	AUC - 0.831^§^ Accuracy - 89.7%^§^
Biron et al. (2020)	Retrospective	Select Candidates for Outpatient Surgery	History / Demographics	4,500 (all ASA)	AUC - 0.77
Polce et al. (2021)	Retrospective	Patient Postoperative Satisfaction	History / Demographics Questionnaires	413 (both ASA and RSA)	AUC - 0.80
Oeding et al. (2023)	Retrospective	Risk of Prosthetic Dislocation	History / Demographics	740 (all RSA)	AUC - 0.71
Gowd et al. (2019)	Retrospective	Postoperative complications	History / Demographics Laboratory Results	17,119 (both ASA and RSA)	AUC - 0.71 Accuracy - 95.4%
Kumar et al. (2020)	Retrospective	Improvement in Multiple Clinical Outcomes^¶^	History / Demographics Questionnaires Physical Exam Findings Imaging Results	4,782 (1,895 ASA, 2,887 RSA)	AUC - 0.875^§^ Accuracy - 92.9%^§^
Rajabzadeh-Oghaz et al. (2024)	Retrospective	Improvement in Multiple Clinical Outcomes^¶^	History / Demographics Questionnaires Physical Exam Findings Imaging Results	1,057 (258 ASA, 799 RSA)	AUC - 0.753^§^ Accuracy - 87.1%^§^
Simmons et al. (2023)	Prospective	Improvement in Multiple Clinical Outcomes^¶^	History / Demographics Questionnaires Physical Exam Findings	243 (43 ASA, 200 RSA)	AUC - 0.841^§^ Accuracy - 89.9%^§^

Studies are denoted by the last name of the first author. ASA, anatomic shoulder arthroplasty; RSA, reverse shoulder arthroplasty; ASES, American shoulder and elbow surgeons; VAS, visual analog pain scale; ROM, Range of Motion; AUC, Area under the curve; MCID, minimal clinically important difference; SCB, substantial clinical benefit. *For each study the Dataset includes the total number of patients included in the analysis, and following this the corresponding number of patients that received either an anatomic or reverse total shoulder arthroplasty is denoted in parentheses. ^†^For each study the Predictive Ability includes the area under the curve, accuracy, and sensitivity if these values were reported. The values are an average of both ASA and RSA if both values were provided in the study. If a study tested multiple different predictive models, the reported values of the model with the highest predictive capabilities is listed here. ^‡^Sensitivity in this study was measured for 3 different subgroups in the study, and this number represents the average of the subgroup sensitivities (0.91, 0.94, 0.98). ^§^In this study, AUC and accuracy were measured for both the MCID and SCB. Only the corresponding values for MCID are listed here. MCID is the smallest change in a treatment outcome that would indicate a clinically significant improvement in the patient’s condition. SCB is the magnitude of improvement in a clinical outcome that reflects a substantial, clearly meaningful benefit from the patient’s perspective. ^¶^These studies used machine learning models to predict numerous clinical outcome measures such as ASES score, UCLA score, SAS score, Constant score, Global Shoulder Function score, visual analog scale (VAS) pain score, active abduction, active forward elevation, and active external rotation. Additionally, AUC and accuracy are listed as the average of the individual AUC and accuracy values calculated for each of the individual clinical outcome measures.

### Measurement tools

AI has also been applied to automated motion analysis and physical assessment in 16 articles (Burns et al., 2018; Darevsky et al., 2023; Darevsky, 2023; Dousty and Zariffa, 2021; Gauci et al., 2023; Gu et al., 2022; Ibara, 2023; Kim et al., 2021; Koyama et al., 2022; Koyama et al., 2021; Lee et al., 2016; Ramkumar et al., 2018; Rostamzadeh et al., 2024; Silver et al., 2006; Takigami et al., 2024; Tsukamoto et al., 2024; Tuan et al., 2022). These studies explored AI models designed to analyze videos or images of body movements including shoulder range of motion, hand gestures, grip strength, and thumb opposition.

Six studies (Darevsky et al., 2023; Gu et al., 2022; Koyama et al., 2021; Ramkumar et al., 2018; Takigami et al., 2024; Tsukamoto et al., 2024) utilized widely accessible devices, such as smartphones and smartwatches, to aid in automated physical examination. These AI models demonstrated high accuracy, exceeding 90% in classifying rotator cuff injuries and nerve dysfunction based on motion analysis. One study used AI-powered pose estimation to measure shoulder internal and external rotation, achieving a correlation coefficient of 0.971 and a mean absolute error of 5.778° compared to standard goniometric measurements (Takigami et al., 2024) (Table 3).

**Table 3:** The tasks and results from the six studies which analyzed AI models’ ability to perform measurements from easily accessible devices such as a smartphone or smart watch.

Study	Smartphone AI task	Dataset	Accuracy
Darevsky et al. (2023)	Measure video recordings of a string-pulling task to classify human patients as having a RC tear	12 participants: 6 patients with RC pathology and 6 healthy volunteers	Accuracy - > 90%
Gu et al. (2022)	Analyze images to detect abnormal hand gestures and classify patients with nerve injury	56 participants (total of 1,344 images): 22 patients, 34 volunteers	Accuracy - > 95% accuracy (all models)
Ramkumar et al. (2018)	Measure shoulder abduction, internal rotation, external rotation, and forward flexion from video recordings	10 participants without shoulder pain performed the arcs of motion for 5 repetitions	Compared to goniometer, the mean differences for the arcs of motion were abduction, −3.7° ± 3.2°; forward flexion, −4.9° ± 2.5°; internal rotation, −2.4° ± 3.7°; and external rotation −2.6° ± 3.4°
Koyama et al. (2021)	Measure thumb opposition using an app to diagnose patients with CTS	63 participants: 36 patients with CTS and 27 healthy patients	Sensitivity - 94% sensitivity Specificity −67%
Tsukamoto et al. (2024)	Analyze 10 s grip and release videos to diagnose patients with CTS	59 participants: 25 patients with CTS, 34 healthy patients	Sensitivity - 89% Specificity - 83% correlation coefficient of 0.68 with severity on nerve conduction studies
Takigami et al. (2024)	Estimate the shoulder joint internal/external rotation angle using pose estimation AI from video recordings	10 healthy volunteers	Correlation coefficient of 0.971 and a MAE of 5.778 when estimating shoulder joint angle from a direct-facing position

Studies are listed according to the last name of the first author. RC, Rotator Cuff; CTS, Carpal Tunnel Syndrome; AI, Artificial Intelligence; MAE, Mean Absolute Error.

### Prosthetic limb applications

UE orthopedics also includes prosthetic devices, which play a significant role for many amputee patients, and optimizing the function and utility of these devices with AI is an emerging topic of research. AI has played a key role in enhancing prosthetic limb control, particularly through surface electromyography (sEMG)-based myocontrol. Among the 16 studies (Atzori et al., 2014; Atzori et al., 2016; Castellini et al., 2009; Edwards et al., 2016; Hahne et al., 2017; Hwang et al., 2017; Jiang et al., 2020; Malešević et al., 2021; Mastinu et al., 2020; Nowak et al., 2023; Olsson et al., 2019; Osborn et al., 2021; Patel et al., 2017; Schmalfuss et al., 2018; Wang et al., 2022; Wang et al., 2020) in this category, many focused on improving real-time prosthesis functionality through AI-driven motor learning and predictive feedback systems (Table 4).

**Table 4:** Outlines for each of the studies relating to the use of prosthetics the study type (prospective/retrospective), the dataset (number of study participants, whether amputee or non-amputee), and the results of the study (short summary of study results).

Study	Type	Dataset	Results
Jiang et al. (2020)	Prospective	15 non-amputees	CNN algorithms can effectively recognize shoulder muscle movements using EMG input information
Wang et al. (2022)	Retrospective	30 non-amputees	EMG input can improve the grasping process for hand prostheses
Hahne et al. (2017)	Prospective	10 non-amputees 1 transradial amputee	EMG input can help refine and improve movements for hand prostheses
Osborn et al. (2021)	Prospective	1 transhumeral amputee	Over the course of 1 year, prosthesis usage and functional metrics improved with a machine learning-based myoelectric pattern recognition algorithm
Nowak et al. (2023)	Prospective	1 transradial amputee	Through use of a machine learning protocol, both objective and subjective hand prosthesis measures improved over a 1-year period
Patel et al. (2017)	Prospective	10 non-amputees	Incorporating proprioceptive, force, and grip measurements into a machine learning algorithm improved myocontrol in hand prostheses
Edwards et al. (2016)	Prospective	4 non-amputees 1 transhumeral amputee	A real-time prediction learning algorithm improved efficiency in tasks with a robotic arm
Castellini et al. (2009)	Prospective	10 non-amputees	A machine learning technique was able to achieve real-time grip posture and required force for hand actions
Atzori et al. (2014)	Prospective	67 non-amputees 11 transradial amputees	This study represents the beginning of a new database of information used to study machine learning methods in hand prostheses
Wang et al. (2020)	Prospective	1 transradial amputee	A machine learning model based on US input performed similarly to one with EMG input hand prosthesis control
Schmalfuss et al. (2018)	Prospective	10 non-amputees 1 transradial amputee	Subjects controlled a hand prosthesis more rapidly and accurately using a hybrid machine learning model with integrating an extra degree of freedom for control
Olsson et al. (2019)	Prospective	14 non-amputees	CNN algorithms can use EMG input to provide versatile and responsive hand control interfaces
Hwang et al. (2017)	Prospective	15 non-amputees 1 wrist-deficient subject (congenital)	Arm positional changes can make it difficult for accurate myoelectric control despite the use of machine learning models
Atzori et al. (2016)	Prospective	11 transradial amputees	Machine learning algorithms using EMG input can lead to better hand prosthesis integration and optimization
Malešević et al. (2021)	Prospective	20 non-amputees	This study represents the beginning of a new database of information used to study machine learning methods of EMG input in hand prosthesis control
Mastinu et al. (2020)	Prospective	3 transhumeral amputees	Prostheses that allow for somatosensory input to the amputee via neural stimulation along with EMG input to machine learning algorithms may lead to better myocontrol and prosthesis functionality

CNN, Convolution neural network; EMG, electromyography. Each study is denoted by the last name of the first author.

**Figure 5:**
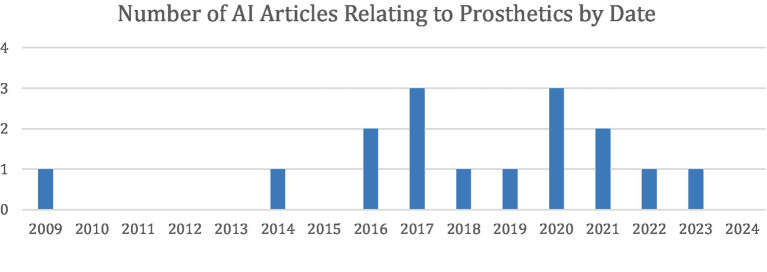
Distribution of the 16 articles under Prosthetic Limb Applications stratified by year of publication.

The first of these articles was published in 2009, and since then, interest in this field has increased significantly (Figure 5). In fact, this was the earliest article included in this review, showing that prosthetics was one of the first areas of interest to implement AI in the UE.

Movements performed by prostheses are performed in an “on/off” fashion, thus rendering coordinated movements with a set amount of force by particular muscles difficult. To overcome this, many prosthesis designs have aimed at incorporating electromyography (EMG) data to allow for more fine-tuned functionality. This is given further power when such input data is processed by way of a machine learning algorithm that can provide real-time feedback and updates as well as learn for future use. AI-driven pattern recognition algorithms have enabled fine-tuned, adaptive myoelectric control, allowing upper extremity amputees to achieve more coordinated, natural movement. Some studies incorporated real-time ultrasound feedback to improve AI-based prosthesis control, achieving accuracy comparable to electromyography-based models (Wang et al., 2020). Others demonstrated that machine learning-enhanced myoelectric control systems could significantly reduce reaction time and improve grip precision in prosthetic hand users (Nowak et al., 2023; Osborn et al., 2021; Patel et al., 2017).

### Intraoperative AI applications

Twelve studies (Bernard et al., 2022; Bockhacker et al., 2020; Cheng et al., 2023; Eslamian et al., 2016; Eslamian et al., 2020; Hein et al., 2021; Kuthiala et al., 2022; Lee et al., 2018; Li et al., 2021; Shafiei et al., 2021; Sühn et al., 2023; Suh et al., 2011) investigated AI’s intraoperative applications, including robotic-assisted surgery, real-time bacterial identification, and automated instrument tracking. Most of these studies are lab-based, with no proof-of-concept in actual surgeries. One study showed that AI-based bacterial identification systems detected osteomyelitis-causing pathogens within five hours, significantly faster and in a less labor-intensive manner than traditional microbial cultures (Bernard et al., 2022). Similarly, another study demonstrated that AI-assisted intraoperative soft-tissue sarcoma classification achieved an accuracy above 85%, outperforming the traditional gold standard of H&E staining frozen sections, which often delays completion of the surgical procedure (Li et al., 2021).

AI-enhanced robotic surgery was explored in three studies (Eslamian et al., 2016; Eslamian et al., 2020; Sühn et al., 2023), showing that autonomous AI-controlled surgical cameras improved visualization, reduced unnecessary movements, and enhanced procedural efficiency and flow. This method was found to be superior to manual camera movement by the surgeon or a trained camera operator. Such technology additionally keeps the surgical instruments in view and avoids unnecessary movement of the camera, preventing inadequate visualization and distraction to the surgeon (Eslamian et al., 2016; Eslamian et al., 2020).

The direct tactile assessment of surface textures during palpation is an essential component of open surgery that is impeded in minimally invasive and robot-assisted surgery. A data generation framework proved accurate (>96%) in using vibro-acoustic sensing to differentiate materials during minimally invasive and robot-assisted surgery. This technology could provide valuable information during procedures such as a total joint replacement or arthroscopy, in which the osteoarthritic cartilage could be identified and graded to help the surgeon plan and make intraoperative decisions (Sühn et al., 2023).

Other intraoperative uses for AI included automated surgeon distraction monitoring (Shafiei et al., 2021), real-time detection of peripherally inserted central catheter (PICC) tips (Lee et al., 2018), segmenting arm venous images (Kuthiala et al., 2022), and gesture-controlled sterile navigation systems. One study evaluating AI-assisted touchless image viewing in the operating room, predicted the hand gestures of eight surgeons with an average of 6.5 years of experience, reaching a 98.94% accuracy in executing the correct task (Bockhacker et al., 2020).

### Clinical decision support tool

AI was utilized as a clinical decision support tool (CDST) in six articles (Bulstra et al., 2022; Daher et al., 2023; Jagiella-Lodise et al., 2024; Rigamonti et al., 2021; Simmons et al., 2022; Yamamoto et al., 2024), meaning they were used in some degree to aid clinical decision-making but did not fall under any of the above categories. These studies focused on diagnostic guidance, treatment planning, and patient education.

Two studies evaluated ChatGPT’s diagnostic capabilities in UE conditions. One study found that ChatGPT correctly diagnosed and recommended appropriate management for 93 and 83% of shoulder and elbow cases, respectively (Daher et al., 2023). Another study assessed ChatGPT’s ability to answer common patient questions related to hand and wrist pathologies, with responses receiving an accuracy rating of 4.83 out of 6 (Jagiella-Lodise et al., 2024).

Another study tested the ability of an AI program to predict scaphoid fractures given elements of a patient’s demographics, history, and physical exam findings without being provided imaging (Bulstra et al., 2022). This machine learning algorithm achieved an area under the receiver operating characteristic curve of 0.77 when predicting the probability of a scaphoid fracture for a retrospective patient cohort. Although accurate, this performance does not exceed that of experienced physicians, who have shown a negative predictive value of up to 96% when predicting scaphoid fractures using a Clinical Scaphoid Score, without the aid of imaging (Pham, 2025). Additionally, this program was able to recommend advanced imaging for patients with a ≥ 10% risk of fracture, yielding 100% sensitivity, 38% specificity, and would have reduced the number of patients undergoing advanced imaging by 36% without missing a fracture.

Another study evaluated how a CDST would help surgeons plan preoperatively whether to perform an anatomic or reverse total shoulder arthroplasty for a patient with osteoarthritis. While this tool did not necessarily direct their decision, it improved their confidence in their own chosen decision (Simmons et al., 2022). Finally, one study discussed the ability of an AI model to analyze gait characteristics from in-shoe wearable monitors to predict distal radius fracture risks (Yamamoto et al., 2024).

These studies are outlined in Table 5.

**Table 5:** Outlines for each of the studies relating to the clinical decision support tools the study task, the dataset (whether real patients, fictional case presentations, survey results, or algorithm responses to questions), and the results of the study (short summary of study results).

Study	Task	Dataset	Results
Bulstra et al. (2022)	Predict scaphoid fractures given patients’ history, demographics, and PE findings and recommend further imaging if needed	Retrospective cohort of 422 patients with radial wrist pain after wrist trauma, 117 confirmed scaphoid fractures	A machine learning model was successfully able to predict scaphoid fractures (0.77 AUC) given patient information and recommend further diagnostic imaging only if needed, reducing overuse of advanced imaging.
Daher et al. (2023)	Provide a diagnosis and treatment plan for patients with UE complaints given patient demographics, PE findings, and imaging results	29 patients with UE complaints	ChatGPT was able to diagnose UE complaints (93%) more accurately than it was able to provide correct treatment recommendations (83%), particularly in situations where multiple treatment options were applicable or depended on patient preference.
Simmons et al. (2022)	Compare surgeon confidence in treatment recommendations without vs. with the help of a CDST	30 orthopedic surgeons with 2 + years of shoulder arthroplasty experience	The addition of CDST results did not dictate or alter treatment recommendations for surgeons but it increased the confidence of their respective surgical recommendations.
Jagiella-Lodise et al. (2024)	Provide accurate information to common questions about orthopedic hand conditions	5 common hand conditions with 12–15 questions each asked to ChatGPT (carpal tunnel syndrome, Dupuytren contracture, De Quervain tenosynovitis, trigger finger, and CMC arthritis)	For basic orthopedic hand conditions, ChatGPT has mostly correct (4.83 out of 6 ± 0.95) but sometimes incomplete (2 out of 3 ± 0.59) responses to questions patients may ask when undergoing self-diagnosis.
Yamamoto et al. (2024)	Estimate patients with DRF using gait features obtained from an in-shoe inertial measurement unit	28 postmenopausal females with DRF, 32 age-matched controls	A machine learning model using in-shoe inertial measurements was able to reasonably predict DRFs (0.740 AUC) in elderly females.
Rigamonti et al. (2021)	Provide correct diagnosis to common sports-related injuries	5 fictional case studies (Concussion, ankle sprain, muscle pain, chronic knee instability (after ACL rupture) and tennis elbow)	All chosen injuries and pathologies were either correctly diagnosed or at least tagged with the right advice of when it is urgent for seeking a medical specialist using a machine learning algorithm; however, with an understanding that user knowledge will affect interpretability of output.

PE, physical exam; AUC, area under the curve; UE, upper extremity; CDST, clinical decision support tool; CMC, carpometacarpal; DRF, distal radius fracture; ACL, anterior cruciate ligament.

### Risk of bias assessment

**Figure 6:**
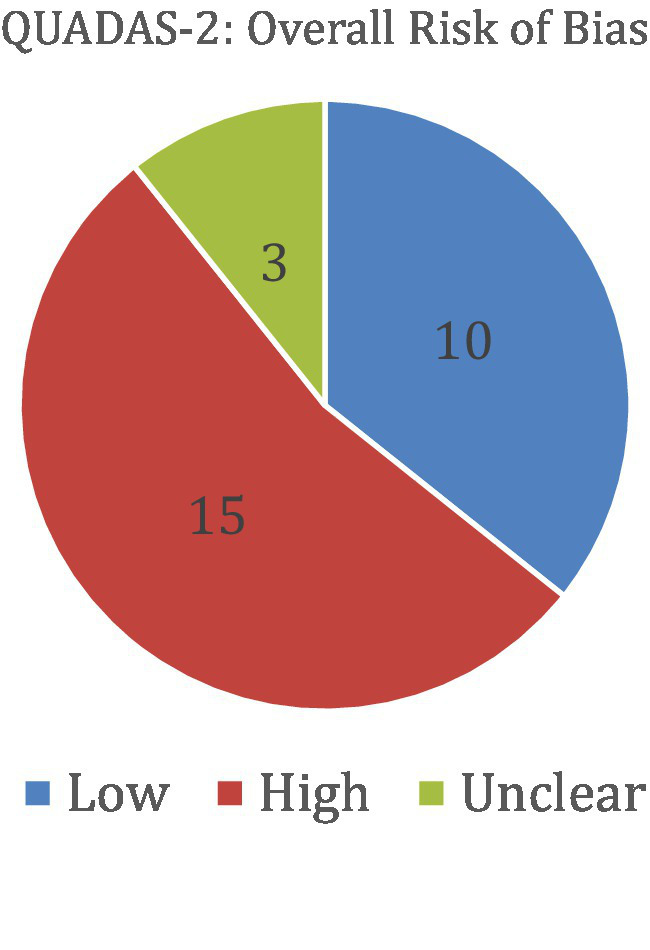
The results of the QUADAS-2 bias analysis regarding whether included studies showed low, high, or unclear overall risk of bias (QUADAS-2).

**Figure 7:**
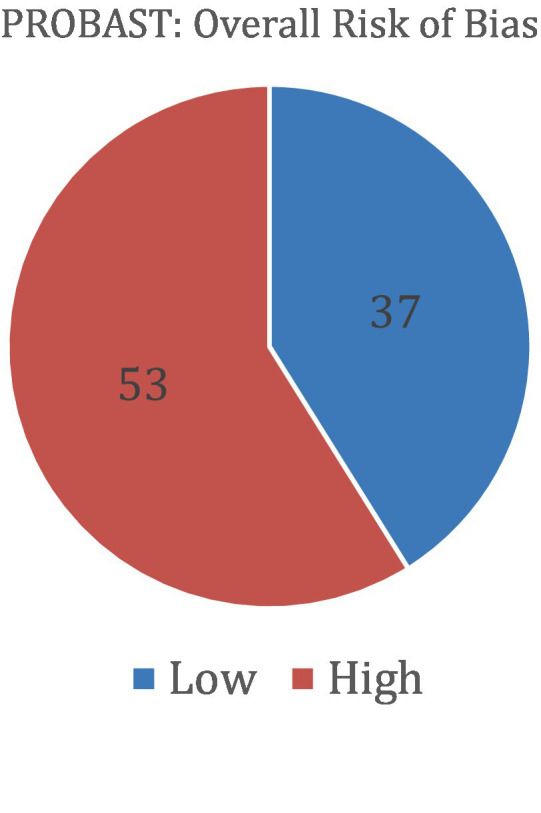
The results of the PROBAST bias analysis regarding whether included studies showed low or high overall risk of bias (PROBAST).

Risk of bias was assessed using the QUADAS-2 tool for diagnostic accuracy studies and the PROBAST tool for prediction model studies. Among the studies evaluated with QUADAS-2 (a total of 28), 15 were judged to have a high overall risk of bias, 10 had a low risk, and 3 had an unclear risk. For studies assessed with PROBAST (a total of 90), 53 demonstrated a high overall risk of bias and 37 had a low risk. No studies in the PROBAST group were rated as having an unclear risk of bias. These assessments provide insight into the methodological quality and potential limitations of the included studies. Table 6 and Figure 6 show the results of the QUADAS-2 analysis, and Table 7 and Figure 7 show the results of the PROBAST analysis.

**Table 6:** The results of the QUADAS-2 bias analysis regarding whether included studies showed low, moderate, high, or unclear risk of bias in the categories of patient selection, index text, reference standard, flow and timing, as well as an overall risk of bias (QUADAS-2).

Study	Patient selection	Index test	Reference standard	Flow and timing	Overall risk of bias
Guermazi et al. (2022)	High	Low	Low	Unclear	High
Droppelmann et al. (2022)	Low	Low	Unclear	Low	Unclear
Yi et al. (2020)	Low	Low	Unclear	Low	Unclear
Guo et al. (2023)	Low	Low	Low	Low	Low
Hahn et al. (2022)	Low	Low	Low	Unclear	Unclear
Chung et al. (2018)	Low	Low	Low	Low	Low
Daher et al. (2023)	High	Moderate	High	High	High
Ro et al. (2021)	Low	Low	Low	Low	Low
Anttila et al. (2023)	Low	Low	Low	Low	Low
Wei et al. (2022)	High	Low	Low	Low	High
Grauhan et al. (2022)	High	Low	High	Low	High
Koyama et al. (2021)	High	Low	Low	Low	High
Feuerriegel et al. (2023)	High	Low	High	Low	High
Benhenneda et al. (2023)	Low	Moderate	High	Low	High
Feuerriegel et al. (2024)	Low	Low	Low	Low	Low
Gauci et al. (2023)	Low	Low	Low	Low	Low
Alike et al. (2023)	Low	Low	Low	Low	Low
Kuok et al. (2020)	High	Low	Moderate	Low	High
Lee et al. (2023)	Moderate	Low	Moderate	Low	Low
Keller et al. (2023)	Moderate	Low	Low	Low	Low
Jopling et al. (2021)	High	Moderate	Low	Low	High
Jagiella-Lodise et al. (2024)	N/A	High	High	Low	High
Jeon et al. (2023)	High	High	Low	Unclear	High
Tuan et al. (2022)	Low	Low	High	Moderate	High
Edwards et al. (2016)	High	Low	Unclear	High	High
Bernard et al. (2022)	High	Low	Unclear	High	High
Simmons et al. (2022)	Low	Low	Moderate	Moderate	Low
Darevsky et al. (2023)	High	Low	Low	High	High

Studies are listed by last name of the first author. N/A (not applicable) is used in where a study did not contain a certain category.

**Table 7:** The results of the PROBAST bias analysis regarding whether included studies showed low, moderate, or high risk of bias in the categories of participants, predictors, outcome, analysis, as well as an overall risk of bias.

Study	Participants	Predictors	Outcome	Analysis	Overall risk of bias
McLendon (2021)	Low	Low	Low	High	High
Kumar et al. (2022)	Low	Low	Low	High	High
Jiang et al. (2020)	Low	Low	Low	High	High
Bulstra et al. (2022)	Low	Low	Low	Low	Low
Kumar et al. (2021)	Low	Low	Low	Low	Low
Roche et al. (2021)	Low	Low	Low	Low	Low
Anttila et al. (2023)	Low	Low	Low	High	High
Dipnall et al. (2022)	Low	Low	Low	Low	Low
Kluck et al. (2023)	Low	Low	Low	Low	Low
Shinohara et al. (2024)	Low	Low	Low	Low	Low
Lu et al. (2021)	Low	Low	Low	High	High
Minelli et al. (2022)	High	Low	High	High	Low
Gu et al. (2022)	Low	Low	Low	Moderate	Low
Biron et al. (2020)	Low	Low	Moderate	High	Low
Polce et al. (2021)	Low	Low	Low	High	High
Ramkumar et al. (2018)	High	Low	Low	High	High
Oeding et al. (2023)	Low	Low	Low	High	High
Kausch et al. (2020)	High	Low	Low	High	High
Gowd et al. (2019)	Low	Low	Low	High	High
Li et al. (2023)	High	Low	Low	High	High
Vassalou et al. (2022)	Low	Low	Low	Low	Low
Kim et al. (2022)	High	Low	Low	High	High
Yang et al. (2024)	Low	Low	Low	Low	Low
Allen et al. (2024)	Low	Low	Low	High	High
King et al. (2023)	Low	Low	Low	High	High
Zech et al. (2023)	Low	Low	Low	High	High
Yoon et al. (2023)	Low	Low	Low	High	High
Shinohara et al. (2023)	Low	Low	Low	High	High
Kang et al. (2021)	Low	Low	Low	High	High
Lee et al. (2024)	Low	Low	Low	High	High
Mert et al. (2024)	Low	Low	Low	High	High
Tsukamoto et al. (2024)	High	Low	Low	High	High
Oeding et al. (2024)	Low	Low	Low	High	High
Ni et al. (2024)	Low	Low	Low	High	High
Zech et al. (2024)	Low	Low	Low	High	High
Takigami et al. (2024)	High	Low	Low	High	High
Suzuki et al. (2022)	Low	Low	Low	High	High
Hoogendam et al. (2022)	Low	Low	Low	Low	Low
Yoon and Chung (2021)	High	Low	Unclear	High	High
Alike et al. (2023)	Low	Low	Low	Low	Low
Simmons et al. (2023)	Low	Low	Low	Moderate	Low
Silver et al. (2006)	High	Low	Moderate	High	High
Giladi et al. (2023)	Low	Low	High	Low	Low
Shinohara et al. (2022)	High	Low	Moderate	High	High
Dousty and Zariffa (2021)	High	Low	Moderate	High	High
Gowd et al. (2022)	Moderate	Low	Moderate	High	High
Hein et al. (2021)	High	Low	Moderate	High	High
Anderson et al. (2023)	Low	Low	Low	Moderate	Low
Burns et al. (2018)	High	Low	Moderate	High	High
Darevsky et al. (2023)	Low	Low	Low	High	Low
Lu et al. (2022)	Low	Low	Low	High	Low
Koyama et al. (2021)	High	Low	Moderate	High	High
Kuthiala et al. (2022)	Moderate	Low	Low	High	High
Ibara (2023)	Low	Low	Low	Low	Low
Wang et al. (2022)	Low	Low	Low	High	High
Georgeanu et al. (2022)	Low	Low	Low	High	High
Hahne et al. (2017)	Low	Low	Low	Moderate	Low
Kim et al. (2021)	Low	Low	Low	High	High
Rostamzadeh et al. (2024)	Low	Low	Low	High	High
Karnuta et al. (2020)	Low	Low	Low	High	High
Osborn et al. (2021)	High	Low	Low	High	High
Nowak et al. (2023)	High	Low	Low	High	High
Sühn et al. (2023)	High	Low	High	Low	Low
Cirillo et al. (2019)	High	Low	High	Moderate	Low
Li and Ji (2021)	High	Low	High	Moderate	Low
Lee et al. (2018)	Moderate	Low	Moderate	Low	Low
Patel et al. (2017)	High	Low	Moderate	Moderate	Low
Digumarthi et al. (2024)	Low	Low	Low	Moderate	Low
Lee et al. (2016)	Low	Low	Low	High	High
Shafiei et al. (2021)	High	Low	High	High	High
Castellini et al. (2009)	Low	Low	Low	Moderate	Low
Atzori et al. (2014)	Low	Low	Low	Low	Low
Wang et al. (2020)	High	Low	Low	High	High
Suh et al. (2011)	High	Moderate	Low	High	High
Schmalfuss et al. (2018)	Moderate	Low	Low	High	High
Yamamoto et al. (2024)	Low	Low	Low	Moderate	Low
Eslamian et al. (2020)	Low	Low	Low	Moderate	Low
Olsson et al. (2019)	Low	Low	Low	Moderate	Low
Hwang et al. (2017)	Low	Low	Low	Moderate	Low
Eslamian et al. (2016)	Low	Low	Low	Moderate	Low
Atzori et al. (2016)	Moderate	Low	Low	Moderate	Low
Bockhacker et al. (2020)	High	Low	Low	High	High
Cirillo et al. (2021)	High	Low	Low	High	High
Li et al. (2021)	High	Low	Low	High	High
Rigamonti et al. (2021)	High	Low	Unclear	High	High
Malešević et al. (2021)	High	Low	Low	High	High
Mastinu et al. (2020)	Low	Low	Low	High	High
Cheng et al. (2023)	High	High	High	High	Low
Rajabzadeh-Oghaz et al. (2024)	Low	Low	Low	Moderate	Low
Kumar et al. (2020)	Low	Low	Low	Moderate	Low

Studies are listed by last name of the first author.

## Discussion

The rapid evolution of AI has reshaped multiple domains of medicine, including orthopedics. While machine learning has been extensively used for over a decade in myoelectric control for upper limb amputees, the past 2 years have witnessed an unprecedented surge in AI applications across UE surgery. This growth reflects both the increasing sophistication of AI models and a growing recognition of their potential to enhance diagnostic precision, streamline surgical workflows, and improve patient outcomes. Our systematic review categorized AI applications into six primary domains: imaging analysis, surgical outcome prediction, intraoperative assistance, measurement tools, prosthetic limb control, and clinical decision support systems (CDSTs).

Among these, AI-driven imaging analysis has shown the most immediate and impactful benefits. AI models now routinely match or exceed human performance in detecting fractures (Chung et al., 2018; Zech et al., 2023; Mert et al., 2024; Suzuki et al., 2022), measuring critical anatomical angles (Minelli et al., 2022; Gu et al., 2022), and identifying soft tissue pathologies (Droppelmann et al., 2022; Guo et al., 2023; Hahn et al., 2022; Kang et al., 2021; Ni et al., 2024). Although few studies (Guo et al., 2023; Mert et al., 2024) showed surgeons capable of outperforming AI, deep learning algorithms have demonstrated higher sensitivity and specificity than experienced clinicians in certain diagnostic tasks, reinforcing their utility in radiographic interpretation. When AI and human performance are clinically integrated together, results improve. For example, Guermazi et al., demonstrated AI-assisted fracture readings increased sensitivity by 10% and reduced reading time (Guermazi et al., 2022). Such results emphasize that AI should not be replacing, rather enhancing clinician performance. AI-driven pre-screening of X-rays could improve radiology efficiency and speed by up to 16 s per image (Guermazi et al., 2022). AI-based measurement tools also provide precise quantifications of range of motion (ROM) (Li et al., 2023; Ramkumar et al., 2018), grip strength (Koyama et al., 2021), and hand posture (Gu et al., 2022) using accessible technologies like smartphones and smartwatches. These advancements offer a scalable, cost-effective means to enhance clinical assessments and facilitate remote patient monitoring.

Preoperatively, AI is increasingly utilized for surgical outcome prediction. Machine learning models can synthesize demographic, clinical, and imaging data to forecast postoperative ROM, complication risks, and patient satisfaction (Biron et al., 2020; Gowd et al., 2019; Kumar et al., 2021; Kumar et al., 2022; Mclendon, 2021; Oeding et al., 2023; Polce et al., 2021; Rajabzadeh-Oghaz et al., 2024; Simmons et al., 2023). Notably, some studies found that AI could achieve similar predictive accuracy using a reduced set of input variables, minimizing the burden of extensive data collection while still delivering actionable insights (Mclendon, 2021). This suggests that AI could streamline clinical workflows and assist in personalized treatment planning, optimizing decision-making without overwhelming surgeons with unnecessary data entry. Additionally, AI implementations continue to expand intraoperatively, with notable advancements in robotic-assisted surgery, real-time microbial identification, automated surgical instrument tracking, and vibro-acoustic sensing technologies capable of assessing cartilage integrity (Bernard et al., 2022; Hein et al., 2021; Sühn et al., 2023). For example, using AI to identify microbial infections could reduce waiting time on results from days to hours, allowing physicians a quicker response to identify and treat infections (Bernard et al., 2022). Such advancements could refine decision-making in joint preservation or arthroplasty procedures.

The ethical implications surrounding AI integration in UE surgery demand consideration. One pressing concern is algorithmic bias: if training datasets lack sufficient representation of minority groups (e.g., racial or ethnic minorities), fracture-detection or surgical-planning algorithms may underperform for those populations, exacerbating existing health disparities. For instance, studies have documented that AI models trained on primarily White patient data perform less accurately on underrepresented groups, leading to potential misdiagnoses or treatment delays (Pham, 2025). Ethical best practices call for inclusive, diverse datasets, regular demographic performance audits, and adoption of fairness-aware algorithm design methods (e.g., reweighting, adversarial debiasing) to ensure equitable care across populations (Pham, 2025). Moreover, AI systems often function as “black boxes,” complicating informed consent and undermining the doctor-patient relationship if neither patient nor clinician can understand the rationale behind AI-driven recommendations (Kumar et al., 2025). Ensuring meaningful transparency, such as explainability reports and shared decision-making frameworks, is essential. Without these safeguards, AI risk reinforcing, rather than reducing, disparities in surgical care.

Integrating AI into UE surgery holds great promise, but significant implementation barriers remain. Regulatory delays, particularly lengthy FDA clearance processes, pose a major hurdle. Only about half of AI-assisted orthopedic devices have undergone dynamic clinical validation, and many remain untested in real-world surgical settings, slowing adoption (Kumar et al., 2025). Training needs represent another critical obstacle. Orthopedic surgeons often lack formal education in AI or data science; moreover, generational divides influence perceived ease of use, with senior surgeons reporting lower familiarity and higher learning effort requirements (Schmidt et al., 2024). Surveys highlight infrastructure limitations—such as lack of institutional support, AI courses, and interdisciplinary collaboration—as persistent constraints, despite growing interest and ethical concerns like explainability and accountability. Finally, there is the question of legal liability. When an AI-assisted diagnosis or treatment is incorrect and leads to an adverse medical outcome, there is debate whether liability should fall on the company that developed the algorithm, the physician who used the tool, or the regulatory agency that approved it (Cestonaro et al., 2023). These intertwined challenges, regulatory bottlenecks, educational gaps, and infrastructural barriers, need to be addressed systematically to enable safe, effective integration of AI into UE orthopedic practice.

The objective of this literature review was to identify the current applications of AI in UE surgery. In order to cover a broad spectrum to this robust topic and find studies which UE surgeons may find interesting, we selected general search keywords. In agreement with the objective of this review, to give the reader a meaningful overview of the broad topic, we conducted this systematic review with clustering of the articles into six groups of thematically related publications. One limitation to our study is publication bias as studies with successful or positive results are more likely to be published. In addition, most of the studies in prosthetics are characterized by small sample sizes, which may limit their clinical relevance. Another limitation is that some studies overlapped into multiple sections. For example, two studies (Minelli et al., 2022; Gu et al., 2022) tested an AI model’s ability to analyze radiographs and measure critical shoulder angles. One study segmented burn images, but also accurately predicted the length of recovery needed based on burn depth (Cirillo et al., 2021). Additionally, one study used AI as a CDST to effectively predict shoulder surgery outcomes (Simmons et al., 2023). To determine which section to label these “overlap” studies, discussion took place between the primary reviewers until a consensus was achieved. A numeric comparison (accuracy, AUC, dataset, sensitivity, etc.) between certain studies took place when feasible, and the results were listed in their respective tables; however, another limitation to our study is that the majority of our sections contained rather unclear boundaries in terms of association to “artificial intelligence” and “upper extremity surgery.” To address this limitation and achieve the objective of this systematic review, we decided to interpret these vague sections in a narrative and qualitative fashion with citation of comparable publications. Although the target audience of our study is primarily medical professionals, a limitation to this study is that our literature search was conducted using only the MEDLINE/PubMed database, which may introduce selection bias. Most of the studies in our review did not report AI tool type, future research could be directed toward investigating the differences between commercial and academic AI algorithms, particularly in terms of performance, scalability, and transparency. Incorporating Explainable AI techniques such as SHAP, LIME, and DeepSHap into future research and application could also be valuable in aiding physicians in their decision-making process.

## Conclusion

In conclusion, AI is reshaping UE surgery by augmenting diagnostic accuracy, enhancing surgical precision, improving prosthetic control, and facilitating personalized predictive modeling. As AI becomes increasingly embedded in orthopedic practice, future efforts should focus on optimizing real-world applications, addressing ethical and regulatory considerations, and fostering AI literacy among both clinicians and patients. AI should complement, rather than replace, physician expertise, necessitating intuitive interfaces, targeted clinician training, and real-time interpretability to foster trust and adoption among orthopedic surgeons. With continued advancements, AI has the potential to revolutionize orthopedic surgery, driving improvements in patient care, surgical efficiency, and clinical decision-making for years to come.

## Data Availability

The original contributions presented in the study are included in the article/supplementary material, further inquiries can be directed to the corresponding author.
